# Drugs Targeting Sirtuin 2 Exhibit Broad-Spectrum Anti-Infective Activity

**DOI:** 10.3390/ph17101298

**Published:** 2024-09-29

**Authors:** Thomas Shenk, John L. Kulp III, Lillian W. Chiang

**Affiliations:** 1 Evrys Bio, LLC, Pennsylvania Biotechnology Center, 3805 Old Easton Road, Doylestown, PA 18902, USA; tshenk@princeton.edu; 2 Department of Molecular Biology, Princeton University, Princeton, NJ 08544, USA; 3 Conifer Point Pharmaceuticals, Pennsylvania Biotechnology Center, 3805 Old Easton Road, Doylestown, PA 18902, USA; john.kulp@coniferpoint.com

**Keywords:** SIRT1, SIRT2, sirtuin, host-targeted agents, antiviral, anti-infective, allostery, epigenetics, host cell metabolism

## Abstract

Direct-acting anti-infective drugs target pathogen-coded gene products and are a highly successful therapeutic paradigm. However, they generally target a single pathogen or family of pathogens, and the targeted organisms can readily evolve resistance. Host-targeted agents can overcome these limitations. One family of host-targeted, anti-infective agents modulate human sirtuin 2 (SIRT2) enzyme activity. SIRT2 is one of seven human sirtuins, a family of NAD^+^-dependent protein deacylases. It is the only sirtuin that is found predominantly in the cytoplasm. Multiple, structurally distinct SIRT2-targeted, small molecules have been shown to inhibit the replication of both RNA and DNA viruses, as well as intracellular bacterial pathogens, in cell culture and in animal models of disease. Biochemical and X-ray structural studies indicate that most, and probably all, of these compounds act as allosteric modulators. These compounds appear to impact the replication cycles of intracellular pathogens at multiple levels to antagonize their replication and spread. Here, we review SIRT2 modulators reported to exhibit anti-infective activity, exploring their pharmacological action as anti-infectives and identifying questions in need of additional study as this family of anti-infective agents advances to the clinic.

## 1. Introduction

Direct-acting anti-infectives are a highly successful therapeutic paradigm. Of 137 approved antivirals (DrugBank version 5.1.12), the vast majority are direct acting, i.e., they target a viral product. Only thirteen are host targeted and, of these, eleven are interferons, which are generally poorly tolerated. Maraviroc [1] is an exception. It targets the HIV-1 co-receptor, C-C motif chemokine receptor 5 (CCR5) and blocks its interaction with the viral gp120 envelope glycoprotein, preventing CCR5-tropic HIV cell entry.

In spite of their wide use, direct-acting antivirals have two major limitations. The first relates to their breadth of activity. Although remdesivir inhibits the RNA-dependent RNA polymerase of multiple RNA viruses [2], direct-acting agents generally target a single virus or virus family. The second limitation of direct-acting agents is that viruses can readily evolve resistance. In contrast to antivirals, direct-acting anti-bacterials, i.e., antibiotics, are generally broad-spectrum agents, with antimicrobial activity against gram-positive and gram-negative organisms. However, antibiotics suffer from the second limitation of direct-acting drugs: microbes evolve resistance. Host-targeted anti-infectives should overcome these limitations of direct-acting agents. Further, and very importantly, given their broad-spectrum activity, approved host-targeted therapeutics can likely be deployed rapidly against emerging pathogens, even before a detailed understanding of the agent is available.

The concept of host-targeted antivirals is not new, and their potential utility has been reviewed recently [3,4,5,6]. These agents target cell functions essential for the virus but dispensable for normal, quiescent host cells, or they target cell systems that potentiate protective, innate and adaptive immune responses. Epigenetic networks control numerous cellular processes that impact viral replication and spread, and have great potential for the discovery and development of host-targeted antivirals [7,8]. These networks include writers, readers, and erasers, and one common type of epigenetic modification is lysine N-ε-acetylation. This modification has been identified on thousands of proteins, which constitute the cellular acetylome [9]. The acetylome modulates chromatin structure and transcriptional activity, protein interactions and localization, metabolic activity, and numerous other cell processes [10]. Histone acetyltransferases (HATs) write the marks by transferring an acetyl group from acetyl-coenzyme A to a lysine residue, bromodomain and extra-terminal proteins (BETs) bind and read modified lysine residues, and histone deacetylases (HDACs; also termed lysine deacetylases, KDACs) erase the marks. All of these activities are druggable [11,12].

## 2. SIRT2 Impacts the Growth of Intracellular Pathogens

KDACs are comprised of two families: Zn^++^-dependent KDACs and NAD^+^-dependent KDACs. The NAD^+^-dependent enzymes are also termed sirtuins (SIRTs). The seven SIRTs (SIRT1–SIRT7) [13] deacylate lysines in protein substrates, transferring the acyl group to the ADP-ribose of NAD^+^, generating nicotinamide and 2′-*O*-acyl-ADP-ribose. Deacetylation is commonly studied, but SIRTs also remove longer acyl chain modifications [14]. Numerous SIRT inhibitors and activators have been described [15].

Multiple SIRTs impact the growth of intracellular pathogens [16]. In some cases, the SIRT supports replication of the pathogen. For example, SIRT1 has been reported to deacetylate the DNA sensor, interferon gamma inducible protein 16 (IFI16), blocking its cytoplasmic localization and its association with stimulator of interferon genes (STING) [17]. As a result, SIRT1 antagonizes the cellular antiviral response against herpes simplex virus type 1 (HSV-1) in human cells. *Listeria monocytogenes* provides another example. SIRT2 is relocalized from the cytoplasm to the nucleus following infection with the pathogen, where it supports replication of the bacteria, at least in part, by reprograming host cell transcription to prevent premature death of the infected cell [18,19]. In other instances, the SIRT antagonizes replication of the pathogen, as reported for SIRT7 in hepatitis B virus-infected cells, where it binds to the viral genome, termed covalently closed circular DNA (cccDNA), through the viral core protein and acts to restrict viral transcription via modulation of the chromatin structure [20]. Similarly, *Mycobacterium tuberculosis* infection downregulates SIRT1 in macrophages and treatment with an SIRT1 activator, resveratrol or SRT1720, reduced intracellular growth of the bacterium [21].

SIRT2 is the most widely studied sirtuin in the context of infection and multiple SIRT2-targeted modulators have been reported to antagonize the replication of one or more intracellular pathogens. These include DNA viruses, such as human cytomegalovirus (HCMV) and hepatitis B virus (HBV); RNA viruses, such as SARS-CoV-2, influenza A, dengue, and human immunodeficiency virus 1 (HIV-1); and bacteria, such as *L. monocytogenes* and *M. tuberculosis* (Table 1). Known SIRT2-selective modulators with anti-infective activity (Table 2) span multiple chemical classes, comprising aminothiazoles (FLS-359) [22], cyanopropenamides (AGK2) [23], and sulfamoyl benzamides (AK-1 and AK-7) [24,25,26]. In addition, modulators inhibiting both SIRT1 and SIRT2 (SIRT1/2) with demonstrated anti-infective activity (Table 2) include aminobenzamides (sirtinol) [27] and thiourea derivates (tenovin-1) [28]. SIRT1/2 modulators have been shown to inhibit several RNA viruses, but it is not yet clear whether modulation of both SIRT1 and SIRT2, or only one sirtuin, or an off-target activity, is critical to the antiviral effect. Additional SIRT2-selective modulators have been described [29,30,31,32,33], some with more potent in vitro and in vivo activities, but this review will focus on the compounds in Table 2, shown to have anti-infective activity.

In sum, SIRT2 modulators can mitigate the growth of multiple intracellular pathogens. So far, there have not been reports of intracellular pathogens resistant to SIRT2 modulation, but it is conceivable that some viruses or intracellular bacteria have evolved, that are yet to be identified, into gene products that interact with SIRT2 in ways that could preclude productive access by SIRT2 modulators (conferring resistance), for example, by degrading the enzyme or locking it in an active confirmation.

Is SIRT2 modulation the basis for the broad-spectrum anti-infective activity of these agents? In some cases, the role of SIRT2 as the pharmacological target has been documented by showing that multiple, structurally distinct SIRT2 modulators inhibit the replication of a pathogen. For example, HCMV replication and spread is inhibited by the SIRT2 modulators FLS-359 (IC_50_, 0.5 µM), AGK2 (IC_50_, 3.4 µM), and AK-7 (IC_50_, 8.2 µM), albeit at different potencies, confirming SIRT2 as a critical target [22]. In other instances, the role of SIRT2 has been verified by documenting similar inhibitory results following pharmacological versus genetic perturbation of SIRT2. In hepatitis B virus (HBV)-infected cells, AGK2 treatment, shRNA-mediated knockdown of SIRT2, or treatment with a SIRT2 dominant negative variant, reduced HBc protein expression, HBV core particle formation, and viral DNA accumulation [35]. These results, coupled with the observation that a second, unrelated SIRT2 modulator, FLS-359, inhibits HBV cccDNA formation and viral gene expression [34], make a compelling case for the role of SIRT2 in HBV infection and establish SIRT2 as a relevant pharmacological target of AGK2. Similar experiments utilizing both AGK2 and SIRT2-specific shRNAs have confirmed the importance of SIRT2 as a target during the intracellular growth of *L. monocytogenes* [18] and *M. tuberculosis* [42]. Of note, both bacterial infections caused the nuclear accumulation of SIRT2, which normally resides predominantly in the cytoplasm, inducing an altered host cell transcriptome that appears likely to support the growth of the pathogens; AGK2 treatment significantly reversed the changes. There are instances where SIRT2 knockdown experiments are not consistent with the results of pharmacological treatments, presumably resulting from selective drug modulation of SIRT2 activities, as discussed below.

As for all drugs, off-target activities can lead to toxicity, but might also contribute to antiviral efficacy. As a case in point, tenovin-1 inhibits the growth of flaviviruses and bunyaviruses [39,40]. In addition to modulating SIRT1 and 2 [28], tenovins inhibit dihydroorotate dehydrogenase (DHODH), a key enzyme in the pyrimidine synthesis pathway and also block uridine uptake in cultured cells [44]. These activities likely contribute to the antiviral activity of tenovin-1.

## 3. Biochemistry of SIRT2 Modulators

### 3.1. SIRT2 Isoforms

Three mRNA splice variants produce three known SIRT2 protein isoforms [45]. SIRT2 isoform 1 (SIRT2.1) is the full-length, 398 amino acid isoform; SIRT2 isoform 2 (SIRT2.2) lacks amino acids 1–37 that are present in the full-length protein; and SIRT2 isoform 5 (SIRT2.5) lacks amino acids 6–76. SIRT2.1 and 2.2 are predominantly cytoplasmic but move between the nucleus and cytoplasm and can accumulate in the nucleus altering the cellular transcriptome under specific physiological conditions, for example, mitosis [46,47] or, as noted above, bacterial infection [18,42]. SIRT2.5 lacks the nuclear export sequence present in SIRT2.1 and 2.2, which is located at amino acids 31–41, and appears to be exclusively nuclear. Purified SIRT2.5 is inactive in biochemical assays, raising the possibility that it requires an as yet unidentified cofactor or that it performs a non-enzymatic role in the nucleus [45]. SIRT2.1 and 2.2 can remove a variety of protein modifications at the ε-position of the lysine side chain (Table 3) [48,49], but most biochemical studies have focused on deacetylation.

### 3.2. SIRT2 Catalytic Mechanism

**Figure 1:**
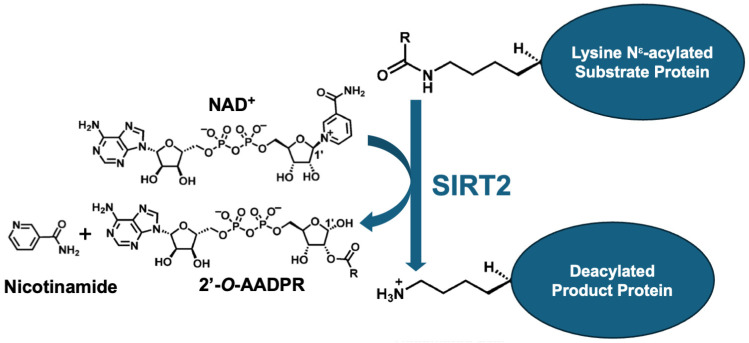
Summary of SIRT2-mediated deacylation. SIRT2 KDAC consumes co-factor NAD^+^ to deacylate a substrate protein, producing the deacylated product protein, nicotinamide, and 2′-*O*-ADP-ribose (2’-*O*-AADPR). R in the substrate protein stands for the acyl chain that can be of varying length, degree of saturation, and charge, along with other chemically diverse substituents.

SIRT2 shares a conserved active site with the seven SIRT family members that catalyze the removal of fatty acyl chains from the acylated lysines of substrate proteins (Figure 1). The catalytic mechanism of SIRT2 deacylation has been delineated in detail [14,55,56,57,58]. Briefly, the reaction begins with the formation of an alkylamidate intermediate between the acyl lysine substrate and NAD^+^, with the release of nicotinamide. A conserved SIRT histidine residue then deprotonates the ribose 2′-OH, which in turn attacks the amidate carbon to generate an intermediate that decomposes to produce 2′-*O*-acyl-ADP-ribose, plus the deacylated lysine. The products produced are the deacylated product protein, nicotinamide, and *O*-acyl-ADP-ribose (Figure 1). Of note, high concentrations of the released nicotinamide can feedback and inhibit the enzyme.

### 3.3. Structures of SIRT2 Modulators Bound to the Enzyme

**Figure 2:**
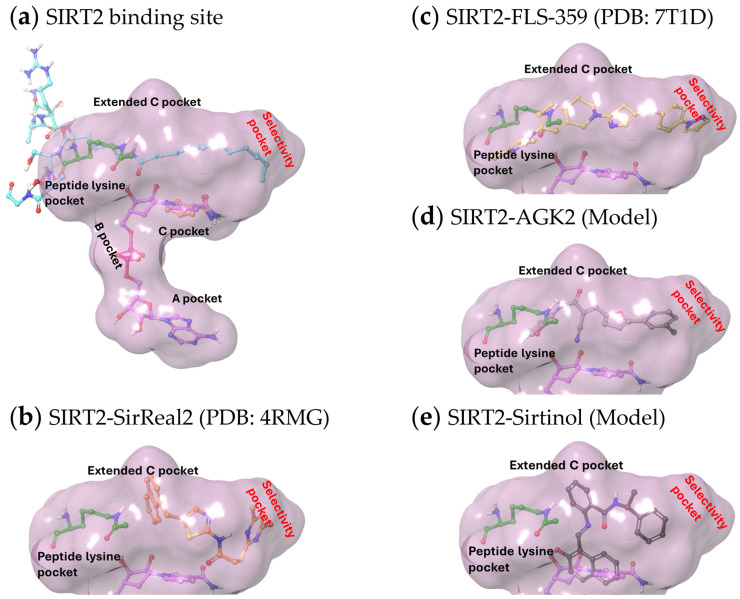
Structures of SIRT2 modulators bound within the SIRT2 active site. (**a**) Binding pockets within the SIRT2 active site (demarcated by a purple cloud) accommodate NAD^+^ (purple carbon atoms, PDB ID 4X3P) as follows: the A pocket binds the adenine ring, the B pocket binds the diphosphate group, and the C pocket binds the nicotinamide ring (orange carbon atoms, PDB ID 6L71). The extended C pocket (EC pocket) binds inhibitors, such as FLS-359 and SirReal2, and acetyl peptides (acetyl-lysine peptide, green carbon atoms, PDB ID 4RMH; myristoyl-lysine peptide, cyan carbon atoms, PDB ID 4X3P). The peptide-binding channel is a solvent-exposed, hydrophobic tunnel that extends from the protein surface to the catalytic site, accommodating acyl lysine residues within substrate proteins. (**b**–**e**) The acetyl peptide (green carbon atoms) was superimposed on the structures, as well as the portion of NAD^+^ sitting within the C pocket (purple carbon atoms), except for (**b**) where the solved crystal structure included NAD^+^. (**b**) Crystal structure of SirReal2 (PDB: 4RMG) and NAD^+^ bound to SIRT2. (**c**) Crystal structure of FLS-359 bound to SIRT2 (PDB ID 7T1D). (**d**,**e**) To dock modulators into SIRT2, crystal structures of the target proteins were retrieved from the Protein Data Bank (PDB) and prepared using the Schrodinger Maestro software (Release 2024-2, Version 14.0). Hydrogens were added, missing residues were added, and the protein was optimized by minimizing its energy through the OPLS_2005 force field in the Maestro protein preparation module. The ligand structures were prepared and the energy was minimized prior to docking using the Maestro Ligprep module. Docking simulations were performed using the Schrodinger Glide module (Release 2024-2). Glide’s extra precision (XP) mode was employed to generate docking poses, with a grid box centered on the FLS-359 ligand (PDB ID 7T1D). Post-docking minimization was performed on all poses generated from the Glide docking. (**d**) Lowest energy docking model of AGK2 bound to SIRT2. (**e**) Lowest energy docking model of sirtinol bound to SIRT2.

X-ray structures have been reported for multiple SIRTs, including SIRT2, and consist of two domains: a smaller domain with a Zn^++^-binding site and a larger domain with a Rossmann fold. The two domains form a clamshell, separated by a groove, that accommodates an acyl lysine peptide substrate. In addition to the acyl lysine binding pocket, the active site has been divided into three sites: A and B interact with the ADP ribose of the cofactor NAD^+^ and C contacts its nicotinamide moiety (Figure 2a). The reaction product, nicotinamide, binds within the C pocket, causing NAD^+^ to bind SIRT2 nonproductively until nicotinamide is released and, thereby, nicotinamide can act as a feedback inhibitor of the enzyme [59]. An additional hydrophobic pocket beside the C site is termed the extended C (EC) site [60] (Figure 2a). This EC pocket accommodates extended acyl moieties, such as myristoyl groups [61] (Figure 2a). The SIRT2 inhibitor, SirReal2 [62], was shown to bind within the EC site (Figure 2b), but its occupancy does not interfere with the binding of either NAD^+^ or nicotinamide. Rather, SirReal2 extends into the substrate binding groove and forms a drug-binding pocket that extends beyond the EC site, causing a rearrangement of the SIRT2 active site (Figure 2b). This selectivity pocket was named as such to recognize the SIRT2-selective nature of the compounds that induce its formation [62,63,64,65]. The X-ray structure determination of FLS-359 (Figure 2c) and Glide docking of AGK2 (Figure 2d) [66] position both antiviral SIRT2-selective compounds in the EC plus selectivity pockets. Interestingly, modeling predicts that sirtinol, which modulates SIRT1 and SIRT2, occupies the SIRT2 selectivity pocket and nicotinamide binding site (Figure 2e).

### 3.4. SIRT2 Modulators Demonstrating Anti-Infective Activity Are Allosteric Partial Modulators

SIRT2 remains partially active in deacetylase assays when treated with saturating amounts of SirReal2, FLS-359, AGK2, or MIND4 [67]. These SIRT2-modulating compounds primarily occupy the EC/selectivity pockets, allowing NAD^+^ to occupy its binding site within the A, B, and C pockets, and an acetyl peptide to occupy the acyl lysine binding pocket and undergo catalysis at a reduced rate. This observation is consistent with an allosteric binding mechanism, a mode of action supported by enzyme kinetic studies [22,62] and movement in the FLS-359/SIRT2 structure [22], compared to the SIRT2 apo structure [68]. The partial inhibition of SIRT2 activity might prove to be advantageous for therapeutic uses, by inhibiting intracellular pathogens, while avoiding host cell toxicity that could arise from complete inhibition of the enzyme.

### 3.5. SIRT2 Partial Allosteric Modulators Are Acyl-Substrate Selective

While FLS-359 and AGK2 inhibit deacetylation, they do not inhibit demyristoylation by SIRT2, consistent with modeling that predicts that FLS-359 is displaced from the enzyme when a myristoyl peptide extends fully into the acyl lysine groove [22]. Thus, these compounds exhibit SIRT2 acyl substrate selectivity [22], a phenomenon that has been described for a variety of enzymes [69]. These compounds inhibit deacylation of some but not all SIRT2 acyl substrates. One of the consequences of substrate selectivity is that genetic knockdown or knockout experiments, which ablate all enzyme activities, might not produce the same biological outcome as compounds that selectively inhibit or activate some, but not all, activities of the target. This has been observed for the FLS-359-mediated inhibition of human cytomegalovirus (HCMV) and influenza A virus growth. Although the SIRT2 modulator inhibits their replication in cultured cells (Table 1), the knockdown of SIRT2 exhibited the opposite effect, modestly increasing the yield of these two very different viruses by several fold [70]. As noted above, multiple SIRT2-selective modulators with very different chemical structures inhibit HCMV growth [22], consistent with the view that their antiviral effect is mediated through SIRT2 and results from the selective modulation of SIRT2 activities rather than the complete ablation of enzyme activity. It is not yet clear how broadly the acyl group selectivity of SIRT2 modulators impacts their antiviral activity across distinct virus families and intracellular bacteria. The identification of specific SIRT2 deacylation activities that support pathogen growth should facilitate the design of a new generation of more efficacious, broad-spectrum antiviral drugs.

## 4. Tolerability and Pharmacology of SIRT2 Modulation

Young SIRT2^−/−^ [71] and SIRT2/3^−/−^ [72] mice are healthy; although they have been reported to develop mammary tumors and other abnormalities associated with advanced age or predisposing treatments [73,74,75,76]. Although these observations raise concerns, they are mitigated by the fact that antiviral SIRT2 modulators, such as FLS-359 and AGK2, are acyl substrate-selective, partial inhibitors and would likely elicit different biological effects than the genetic knockout of SIRT2. Further, the acute nature of many infections should minimize the duration of the treatment; and the long-lasting antiviral effect of FLS-359, following the treatment of HCMV-infected cells, raises the possibility that dosing schedules can be refined to minimize drug exposure [22].

The presumed barrier to drug resistance of host SIRT2-targeted molecules also suggests that anti-infective dosing can be minimized. Direct-acting anti-infectives are generally administered at high multiples of their IC_90_ [77,78]. For example, for the influenza neuraminidase inhibitor, oseltamivir carboxylate, the IC_50_ is 0.7–2.2 and 0.2–0.6 nM against the widely circulating influenza A strains, H5N1 and H3N2, respectively [79]. The standard, twice daily, 75 mg adult dose, achieves a minimum oseltamivir plasma concentration of ~330 nM, greater than 50 times the IC_90_ [80,81]. This dosing schedule ensures the effective distribution of antiviral drug concentrations to infected tissues and mitigates the evolution of viral resistance. Since pathogens are not likely to become resistant to host-targeted agents, this class of anti-infectives might be used in clinical doses to cover lower multiples of their IC_90_ to achieve a therapeutic window. This potential feature of SIRT2 modulators can be explored in animal models to find a window to achieve anti-infective effectiveness, while minimizing toxicity.

FLS-359 PK was evaluated in BALB/c mice [22]. Following a single 50 mg/kg p.o, the drug exhibited an ~6 h plasma half-life, reaching a maximal plasma concentration (C_max_) of 89 µM. The favorable half-life and C_max_ resulted in good exposure, with an AUC of 713 µM·h/mL. No weight loss or adverse clinical signs were observed after 14 days of dosing at 50 mg/kg b.i.d. The antiviral activity of FLS-359 was tested in two humanized mouse models of HCMV infection, namely the gel foam model, in which human fibroblasts growing in a collagen matrix are implanted into immunodeficient mice [82,83], and the lung only model [84,85], where human lung tissue is implanted. FLS-359 reduced virus production in both models [22].

AGK2 administered at 20–82 mg/kg i.p. reduced pathogen load after *M. tuberculosis* challenge in BALB/c mice [42], HSV-1 challenge in C57BL/6 mice [37], or in HBV transgenic C57BL/6 mice [36]. AK-7 administered at 15 mg/kg i.p. reduced the bacterial load after *S. typhimurium* challenge in C57BL/6 mice [43].

## 5. Anti-Infective Mechanisms of SIRT2 Modulators

SIRT2 impacts numerous cellular functions, ranging from innate defenses to transcription to metabolism, providing many opportunities for SIRT2 modulators to influence the course of an infection. Indeed, it seems likely that any pathogen inhibited by a SIRT2 modulator will likely be impacted by multiple SIRT2-regulated mechanisms. Many cell-autonomous consequences of SIRT2 modulation have been described in the context of cancer and have been reviewed recently [86,87,88,89,90].

### 5.1. Microtubule Activity

A signature feature of cytoplasmic SIRT2.1 and SIRT2.2 [91], together with KDAC6 [92], is their ability to deacetylate the K40 residue of α-tubulin. AGK2 primarily causes hyperacetylation of perinuclear microtubules, in contrast to the KDAC inhibitor tubacin, which increases α-tubulin acetylation throughout the cell, suggesting that the activities of SIRT2 and KDAC6 are influenced by the structural environment of the target microtubules [93]. This activity can have major consequences for intracellular pathogens [94,95], controlling the dynamics of intracellular movements of the invader’s components. In the case of HCMV, microtubule activity is critical for the structure and function of the perinuclear assembly zone [96], where capsids are assembled into virions. The potential consequences of SIRT2 modulation on the function of this virus-induced organelle, as well as the infectious process of other viruses and intracellular bacteria, await study.

### 5.2. Innate Defense

The cyclic GMP-AMP synthase (cGAS), the stimulator of interferon genes (STING) pathway, is a key element of innate immunity [97]. Moreover, cGAS is a cytoplasmic receptor that recognizes inappropriately localized DNA and triggers the production of cyclic GMP-AMP (cGAMP) that induces TANK-binding kinase 1 (TBK1)-mediated phosphorylation of interferon regulatory factor 3 (IRF3). IRF3 then moves to the nucleus and activates the transcription of interferon-stimulated genes (ISGs), including the type 1 interferon. The stress granule protein, GTPase-activating protein SH3 domain-binding protein 1 (G3BP1), binds to cGAS, promoting DNA binding and its activation [98,99]. Depletion of G3BP1 reduces interferon production via the cGAS pathway. SIRT2 has been reported to negatively regulate the cGAS–STING pathway, by deacetylating G3BP1 at K257, K276, and K376, and blocking the G3BP1–cGAS interaction [37]. Consistent with the role of SIRT2, AGK2 reduced HSV-1 production in cultured cells several fold, and also reduced the virus load and extended the survival of the mice in a lethal model of HSV-1 infection, increasing the expression of ISGs. Importantly, the cGAS inhibitor, RU.521, counteracted the beneficial effect of AGK2 in mouse experiments [37], confirming the mechanism. It will be very interesting to learn whether the effect of SIRT2 on the cGAS–STING response broadly inhibits the growth of DNA viruses in cell culture and animal models.

In contrast to inhibiting the cGAS–STING pathway, SIRT2 has been reported to promote type 1 interferon signaling. This was explored by testing SIRT2^−/−^ versus SIRT2^+/+^ mouse fibroblasts or by evaluating SIRT2-specific shRNA-treated SIRT2^+/+^ mouse cells. SIRT2 deacetylates cyclin-dependent kinase 9 (CDK9), enhancing its ability to phosphorylate and activate the signal transducer and activator of transcription-1 (STAT1). For most interferon-responsive genes, the inhibitory effect of SIRT2 depletion was partial and SIRT2 modulators were not tested. This result raises the possibility that SIRT2 modulators may differentially modulate the cGAS–STING response, versus signaling through the type 1 interferon receptor. It is not yet clear whether this could antagonize the anti-infective activities of SIRT2 modulation, or whether it might prove beneficial and reduce inflammation associated with the response to infection.

G3BP1 also modulates the growth of numerous RNA viruses via multiple mechanisms [100], but the role of SIRT2 in these activities has not yet been evaluated. Of note, G3BP1 increases type I interferon production by the retinoic acid-inducible gene I (RIG-I) RNA receptor in response to viral dsRNA [101,102], raising the possibility that SIRT2 modulators may prove to enhance the innate immune response to both DNA and RNA viruses.

### 5.3. Intracellular Signaling

**Figure 3:**
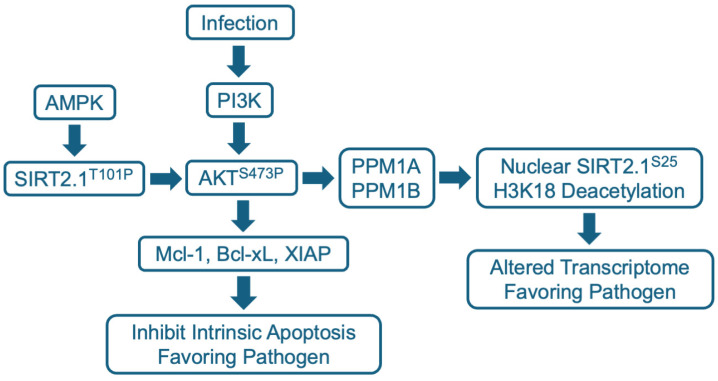
SIRT2.1 modulation of the AKT pathway during infection. AKT is activated by the phosphorylation of S473 via the PI3K pathway. SIRT2.1 is stimulated to bind to AKT via AMPK-mediated phosphorylation at SIRT2.1 T101. SIRT2.1 T101P binds to AKT through its PH and catalytic domains, and presumably deacylates a target lysine within AKT to stimulate maximal interaction with PI3K, leading to phosphorylation and activation of AKT. Activated AKT potentially has many consequences in the cytoplasm, including induction of anti-apoptotic Bcl-2 family members such as Mcl-1, Bcl-xL, and XIAP. The PP2C family members PPM1A and PPM1B, which are present in both cytoplasm and the nucleus, can dephosphylate SIRT2.1 S25, which then accumulates in the nucleus. Nuclear SIRT2 then deacetylates H3K18 and alters the cell’s transcriptome to favor growth of the pathogen.

The AKT (aka protein kinase B, PKB; a serine/threonine kinase) pathway has been studied in the context of SIRT2 modulation during infection (Figure 3). Its activation and regulation are complex [103,104]. Multiple upstream cell sensors, such as receptor tyrosine kinases, activate specific phosphoinositide 3-kinase (PI3K) isoforms to produce phosphatidylinositol 3, 4, 5-triphosphate (PIP3), which then serves as a plasma membrane docking site for AKT and pyruvate dehydrogenase 1 (PDK1) via their pleckstrin homology (PH) domains. PKD1 phosphorylates AKT at T308, an activating modification, and mTORC2 phosphorylates AKT at S473 to achieve full activation.

SIRT2 binds to AKT through its PH and catalytic domains and has been shown to drive its maximal activation in the context of insulin signaling [105] and hepatocellular carcinoma cells [106]. Phosphorylation of SIRT2 at T101 by AMP-activated protein kinase (AMPK) stimulates SIRT2–AKT binding. Reduced SIRT2 activity reduces AKT activation, whereas SIRT2 overexpression increases AKT activation. As noted above, AKT activation involves T308 and S473 phosphorylation, and the SIRT2 modulator, AGK2, prevented AKT hyperphosphorylation at S473 in response to insulin or the epidermal growth factor (EGF) [105]. The basal level of activated AKT S473P was not reduced, suggesting that elevated, but not basal, AKT activity was blocked by the SIRT2 modulator.

AKT was first described as an oncogene, v-AKT, a gag–AKT fusion protein encoded by the AKT8 murine retrovirus [107]. The acquisition of the kinase into a viral genome was the first indication of its importance for viral replication. Multiple viruses have been shown to activate AKT [108], including DNA viruses, such as herpesviruses [109], human papillomavirus [110], and hepatitis B virus [111]; RNA viruses like influenza [112] and SARS-CoV-2 [113]; and the retrovirus HIV-1 [114,115]. The PI3K/AKT pathway impacts numerous cell processes, including RNA processing and translation, metabolism, cell proliferation, and cell survival and, in many cases, activated AKT supports viral replication.

The activation of AKT can extend survival, preventing the premature death of virus-infected cells, allowing a virus to complete its replication cycle and spread. The herpesvirus, HCMV, provides an example, where SIRT2 has been shown to play a key role in this process [116]. HCMV establishes a quiescent infection in human monocytes, during which viral gene expression is suppressed. This quiescent state enables the virus to travel with monocytes throughout the infected individual, before eventually reactivating when the monocyte differentiates into a macrophage [117,118]. However, the lifespan of a monocyte is limited, they survive only for 48–72 h before undergoing apoptosis. HCMV infection blocks this process, markedly extending the lifespan of quiescently infected monocytes [119]. This is accomplished as the HCMV virion engages the monocyte. It induces phosphorylation of AKT at S473 [120], activating the kinase, which in turn upregulates the expression of the anti-apoptotic Bcl2 family member, myeloid cell leukemia sequence 1 (Mcl-1) [119] (Figure 3). The virus then establishes a long-lived quiescent infection in the monocyte. The SIRT2 modulator FLS-359 can block HCMV-induced phosphorylation of AKT at S473 in primary human monocytes and prevent the downstream accumulation of Mcl-1 [116]. This leads to the death of the infected monocytes and, therefore, has the potential to block a key element of HCMV persistence and spread in infected individuals. SIRT2 modulation of AKT activation has also been shown to play a key role in the expression of HBV-coded RNAs, and this is discussed below.

Like SIRT2, SIRT1 has been reported to stimulate the activation of AKT [121]. Acetylation of two lysine residues in the AKT PH domain, K14 and K20, can inhibit AKT binding to PIP3, and SIRT1 can deacetylate AKT, promoting its interaction with PIP3 and activating the kinase. SIRT1^−/−^ mouse fibroblasts showed reduced insulin-like growth factor 1 (IGF-1)-mediated activation of AKT compared to SIRT1^+/+^ cells. It is likely that SIRT1 and SIRT2 act similarly to deacetylate the AKT PH domain and promote its activation, and the basal AKT activity observed in cells treated with the SIRT2-selective inhibitor AGK2 [105], might result from the continuing action of SIRT1. It is possible that dual SIRT1/2 modulators, such as sirtinol and tenovin-1, may prove to more severely inhibit AKT activation and, therefore, more stringently control AKT-dependent antiviral mechanisms. Of course, potentially increased antiviral activity resulting from greater inhibition of AKT activation might be accompanied by increased toxicity.

### 5.4. Host Cell and Viral Transcription

SIRT2.1 and SIRT2.2 reside predominantly in the cytoplasm, but they can be relocalized to the nucleus during infection via an AKT-dependent process (Figure 3). This was first shown for the intracellular bacterium, *L. monocytogenes* [18]. The *L. monocytogenes*-coded virulence factor, InlB, induced the nuclear accumulation of SIRT2. The nuclear localization was blocked by AKT inhibitors or a dominant negative p85 AKT regulatory subunit. In the nucleus, SIRT2 deacetylated H3K18 at transcriptional start sites and altered the host transcriptome: 158 host cell genes were activated and 272 were repressed. Importantly, the SIRT2 modulator, AGK2, substantially blocked the infection-induced changes. Further, although AGK2 was not toxic to free growing bacteria, it reduced bacterial growth in infected cells in culture and in mice. Presumably, nuclear SIRT2 imposes a host cell transcriptional program that favors the growth of *L. monocytogenes* and a block to this program by AGK2 inhibits growth of the pathogen. Exactly how AKT promotes nuclear localization of SIRT2.1 remains unclear. However, a subsequent report [122] showed that infection induces a complex between SIRT2 and the phosphatases PPM1A and PPM1B that localizes to chromatin and leads to the dephosphorylation of SIRT2 S25 (Figure 3), which is required for its nuclear accumulation and H3K18 deacetylation activity.

A similar SIRT2 nuclear localization and altered host cell transcriptome has been reported for *M. tuberculosis*-infected macrophages, where AKT is activated [42]. Again, AGK2 treatment inhibited AKT activation, substantially reversed the altered transcriptional program, and inhibited the intracellular growth of the pathogen. A major problem with the treatment of *M. tuberculosis* using currently available, direct-acting drugs is that the pathogen readily evolves drug resistance. Host-targeted AGK2 inhibited the growth of drug-sensitive, multiple drug-resistant, and extensively drug-resistant *M. tuberculosis* isolates and it cooperated with the standard-of-care, isoniazid, to inhibit *M. tuberculosis* infection in mice [42], emphasizing the potential utility of SIRT2 modulators in the clinic.

Not surprisingly, in addition to the effects on cellular transcription, SIRT2 modulators can target the expression of genes coded by the infecting agent. SIRT2.1 is increased following HBV infection; the overexpression of SIRT2.1 increased the levels of viral RNAs in infected cells, as well as the activity of all HBV promoter/enhancer elements in luciferase reporter assays, and AGK2 reduced the expression of viral RNAs [35]. AKT was activated (T308P and S473P) via its interaction with SIRT2 and AGK2 inhibited the activation, suggesting that AKT could play a similar role in the viral infection as documented for the bacterial infections discussed above. A similar inhibitory effect on HBV RNA accumulation and promoter activity was observed for FLS-359; in this study, the SIRT2 modulator was also shown to inhibit the establishment of cccDNA at a point after the virus had entered the cell, when the drug was present at the start of the infection [34]. In another study, FLS-359 inhibited the expression of all the temporal classes of HCMV mRNAs [22], similar to the effect on HBV. The potential translocation of SIRT2.1 and/or SIRT2.2 to the nucleus has not been reported for these viral systems.

One might have anticipated an opposite effect of SIRT2 modulators on viral transcription if they are acting to inhibit nuclear SIRT2, i.e., that treatment with AGK2 and FLS-359 would lead to hyperacetylation of viral chromatin, a state that generally favors active transcription [123]. Perhaps an indirect mechanism, such as the enhanced expression of a cell-coded repressor, inhibits viral transcription. In this regard, p53 has been reported to bind HBV enhancer regions and reduce HBV transcriptional activity [124]. Moreover, p53 can activate or repress transcription [125] and its DNA-binding activity is modulated by the acetylation of its C-terminal lysines [126]. The SIRT1/2 modulator, sirtinol [127,128], as well as two related SIRT2 modulators, AEM1 and AEM2 [129], induce the hyperacetylation of p53 at K382 within its C-terminal domain. The hyperacetylation of p53 in its C-terminus enhances its DNA-binding activity, suggesting that SIRT1/2 or SIRT2 modulators could facilitate the binding of p53 to HBV enhancers in its inhibitory mode, as has already been proposed [130]. Further analysis is needed to ascertain whether p53 contributes to the inhibition of HBV and HCMV transcription by SIRT2 modulators.

### 5.5. Central Carbon Metabolism and Lipid Metabolism

SIRT2 regulates many aspects of metabolism [49,131,132,133], including enzymes that drive glycolysis, the TCA cycle, oxidative phosphorylation (OxPhos), and lipid synthesis. Multiple viruses also induce aerobic glycolysis and OxPhos [134]. So, in this instance, SIRT2 modulators might support aspects of viral replication; although it is possible that the combination of SIRT2 modulation plus viral infection could lead to the high consumption of glucose and the resulting buildup of lactic acid in the microenvironment of infected cells could be detrimental to viral replication. Many viruses also modulate lipid synthesis, for example, the enveloped DNA virus, HCMV, and the “quasi-enveloped” hepatitis A virus [135,136] induces the synthesis of and requires very long-chain fatty acids. Fatty acid synthesis starts with the export of citrate, produced in the TCA cycle, from the mitochondria to cytoplasm, where it is converted to acetyl-CoA and oxaloacetate by ATP citrate lyase (ACLY), and then the acetyl-CoA is converted by acetyl-CoA carboxylase into malonyl-CoA. This is the first committed step in fatty acid synthesis. SIRT2 deacetylates and inhibits ACLY, reducing de novo synthesis of fatty acids and, again, SIRT2 modulators would be expected to support fatty acid synthesis. As a deeper understanding of the role of SIRT2 in metabolism develops, potential antiviral mechanisms of SIRT2 modulators are likely to emerge. In this regard, it is intriguing to note that the treatment of HCMV-infected cells with an elongase 7 inhibitor to block very long-chain fatty acid synthesis [137] and the treatment of infected cells with the SIRT2 modulator, FLS-359 [22], both induce the production of virus particles that are not infectious. Conceivably, the treatments produce the same outcome because they both block the production of very long-chain fatty acids and alter the lipid composition of the virion.

### 5.6. Acetylation of Viral Proteins

In a study of HCMV-coded protein acetylation [138], 32 acetylated lysine residues were identified in virus-coded intracellular and/or virion proteins. Acetylated pUL26 K203 was detected in the intracellular and virion pUL26 protein. The function of the modification was explored by generating viruses carrying an acetyl lysine mimic, pUL26 Q203, or a charge mimic, pUL26 R203. The acetyl lysine mimic reduced the virus yield by several fold, whereas the charge mimic enhanced the yield, suggesting that SIRT2 modulators might reduce HCMV growth, in part, by enhancing the acetylation of virus-coded pUL26. If this is the case, then the virus could potentially evolve resistance to this mode of inhibition via the acquisition of a pUL26 mutation that abrogates inhibition by a SIRT2 modulator. Acetylated lysines have been identified in proteins encoded by a variety of other viruses [139], so SIRT2 modulators might act directly on viral proteins, i.e., as direct-acting antivirals, in multiple instances. If this proves to be a significant mode of antiviral activity, then it might prove possible to isolate viruses resistant to SIRT2 modulators. However, this seems unlikely, since multiple additional host cell-targeted antiviral activities would presumably also be at play. Further, in the specific case of HCMV pUL26, the modest effect of the acetylation mimic on the virus yield (several fold) cannot account for the ~10,000-fold reduction in HCMV infectivity, following treatment with FLS-359 [22].

Given the plethora of SIRT2 targets within an infected cell, it seems likely that multiple mechanisms cooperate to inhibit viral growth in response to SIRT2 modulators, with some mechanisms exerting a greater anti-infective effect than others, depending on the pathogen and cell type.

## 6. Potential Therapeutic Utility of SIRT2 Modulators as Anti-Infective Agents

### 6.1. Combined Cell Autonomous Effects of SIRT2 Modulation

The antiviral mechanisms described above can presumably generate a broad-spectrum antiviral state, perhaps with different SIRT2-mediated mechanisms impacting and controlling different pathogens. This can be highly beneficial in multiple clinical applications. Immunosuppressed transplant patients provide a prime example. These patients are at risk from heightened susceptibility to adventitious pathogens in donor tissues or their environment that include herpesviruses, polyomaviruses, hepadnaviruses, and emerging viruses [140,141,142], several of which are already known to be inhibited by SIRT2 modulators (Table 1). Broad-spectrum activity, combined with a block to viral resistance, has the potential to improve outcomes in this patient population.

And, when the next pandemic begins, a range of broad-spectrum SIRT2 modulators can immediately be taken off the shelf, tested, and potentially deployed to treat the new threat. This could benefit patients, months or even years before safe and effective direct-acting antivirals can be produced, while markedly reducing the cost of new drug development.

The broad-spectrum feature of SIRT2 modulators is not new to the antibiotic world, but host targeting should markedly, if not completely, reduce the burden of bacterial drug resistance.

### 6.2. Immune Modulation

SIRT2 has been described as a master regulator of T cell metabolism [131,132]. In these cells, SIRT2 interacts with eight of ten glycolytic enzymes and four of eight TCA cycle enzymes, and SIRT2 restrains aerobic glycolysis and oxidative phosphorylation (OxPhos) during T cell activation and maturation. Most of these proteins were hyperacetylated in SIRT2^−/−^ cells, where glycolysis and OxPhos were elevated. SIRT2^−/−^ T cells displayed increased proliferation, survival, and effector functions, such as interferon-γ production [131]. Consistent with these results, the SIRT2 modulators, AGK2 and thiomyristoyl (TM), increased aerobic glycolysis, OxPhos, and interferon-γ production in human CD3^+^ T cells, and SIRT2 modulation also enhanced the cytotoxic activity of human tumor infiltrating lymphocytes (TILs) versus their autologous tumor cells. Finally, consistent with the in vitro results, transplanted SIRT2^−/−^ CD8^+^ T cells inhibited the growth of the immunologically cold murine B16F10 melanoma model [131]. All of this to say, genetic ablation or the modulation of SIRT2 can activate T cells, even in the tumor microenvironment, where their function is normally suppressed.

The impact of SIRT2 modulators on T cell activation suggests their potential utility as cancer therapeutics, but is there also an opportunity for the treatment of infections? Chronic HBV infection usually exhibits weak virus-specific T cell reactivity [143]. This state of “T cell exhaustion” is characterized by poor effector cytotoxic activity, limited cytokine production, and limited expression of multiple inhibitory receptors, such as the programmed cell death-1 (PD-1) receptor, and metabolic reprogramming has been proposed as a potential solution to the exhausted T cell phenotype [144]. Perhaps the effector activities of exhausted CD4^+^ and CD8^+^ T cells in chronic HBV infection will respond to treatment with SIRT2 modulators or a combination of the SIRT2 modulator plus the PD-1 inhibitor. If so, this could potentially contribute to resolution of the disease.

The SIRT2 modulator AGK2 has also been shown to enhance the efficacy of the Bacille Calmette–Guerin (BCG) tuberculosis vaccine via its impact on memory T cells [145], consistent with earlier work showing that these cells depend on glycolysis and OxPhos for energy [146] and modulating energy metabolism facilitates the formation and maintenance of CD8^+^ memory T cells [147]. T helper cell function, memory, and cytotoxicity, very likely play important roles in optimal and long-lived immunity [148]. Given their ability to manipulate the T cell response, SIRT2 modulators have the potential to contribute to vaccinology as a broadly effective class of adjuvants.

### 6.3. Inflammation

The resolution of bacterial and viral infections can be complicated by severe inflammation. SIRT2 deacetylates NFKB p65 K310, and the hyperacetylation of K310 in SIRT2^−/−^ cells increases the expression of a subset of NFKB-responsive genes [149]. Since NFKB controls the expression of numerous genes involved in immune and inflammatory responses, genetic or pharmacological modulation of SIRT2 might be predicted to induce inflammation. Consistent with this view, bone marrow-derived murine macrophages treated with lipopolysaccharide (LPS; Toll-like receptor (TLR) 4 ligand), triacylated lipopeptide (Pam3CSK4; TLR1/2 ligand), or the di-nucleotide CpG (TLR9 ligand) produced large amounts of proinflammatory TNF, IL6, and IL12-p40, and treatment with either of the SIRT2 modulators, AK-7 or AGK2, at the time of TLR stimulation failed to reduce the levels of the cytokines [150]. Similarly, relative to wild-type controls, SIRT2^-/-^ mice treated with LPS accumulated hyperacetylated NF-KB and showed increases in pro-inflammatory cytokines. Further, in a model of traumatic brain injury, the SIRT2 modulator AK-7 increased K310 acetylation and upregulated pro-inflammatory cytokines, exacerbating neuroinflammation [151].

However, there are also reports arguing that the manipulation of SIRT2 expression and activity can reduce inflammatory responses. Relative to SIRT2^+/+^ mouse macrophages, LPS-treated SIRT2^−/−^ macrophages exhibited reduced expression of the inflammatory mediator, inducible nitric oxide synthase (iNOS), reduced the production of reactive oxygen species, and reduced NF-KB activation [152]. Similarly, rat microglial cells treated with the SIRT2 modulator AGK2, prior to LPS exposure, exhibited significantly reduced production of the highly inflammatory lipid, prostaglandin E2 (PGE_2_) [153]. AGK2 treatment also suppressed the expression of LPS-induced inflammatory cytokines (iNOS, TNF-α, and IL-1β) in murine microglial cells [154]. As a side issue, treatment with the SIRT1 inhibitor, EX527, also inhibited LPS-induced PGE_2_ production [153].

In sum, the role of SIRT2 in inflammation is likely tissue specific and context dependent [155], and the impact of SIRT2 modulators on infection-associated inflammation remains unresolved.

### 6.4. Sepsis

“Sepsis is defined as life-threatening organ dysfunction caused by a dysregulated host response to infection” [156]. Its pathogenesis includes hyperinflammation; activation of coagulation, vascular epithelium, and complement; and immune suppression [157]. Sepsis progresses in two phases: an early, acute phase, characterized by hyperinflammation [158] and a late, hypoinflammatory phase with the depletion of immune cells [159,160]. Bacteria, viruses, fungi, and parasites can all cause sepsis; although there are differences among the sepsis syndromes induced by the different classes of pathogens [161].

Several mouse studies employing the cecal ligation and puncture (CLP) model of sepsis suggest that SIRT2 modulators have potential therapeutic benefits in sepsis. In this model, a portion of the cecum is ligated and then punctured. Puncture results in polymicrobial peritonitis, bacteremia in the blood, septic shock, and multi-organ dysfunction, followed by death. The CLP model is the gold standard in terms of rodent sepsis models [162]; although there are some issues, such as young healthy mice being the subjects, whereas humans are generally older with co-morbidities; laboratory mice do not have a normal microbiome and the mouse model usually plays out over a considerably shorter timeframe than human disease [163,164]. Older mice are more susceptible to CLP than younger mice [165].

Using C57BL/6J mice, a single i.p. dose of AGK2 (82 mg/kg, n = 9) or vehicle alone was administered 2 h prior to initiation of the CLP, and the SIRT2 modulator improved survival to 55.6% versus 0% for the vehicle alone at 10 days post-procedure [166]. This study evaluated the effect of AGK2 on the development of the early, hyperinflammatory phase of sepsis, as the drug was administered before the initiation of the CLP, and it raises the possibility that SIRT2 modulation might be beneficial if administered during the early, hyperinflammatory phase of sepsis.

The effect of SIRT2 modulation on sepsis using the CLP model was also evaluated in obese (ob/ob; leptin deficient) mice [167]. LPS tolerance, assayed by leukocyte adhesion in the microvasculature, was used to monitor the stage of sepsis. Hyperinflammation peaked at 6 h and decreased at 12 h through 7 d post-CLP, and SIRT2 expression in small intestine cells increased during the hypoinflammatory stage. Ob/ob mice were treated with AK-7 (40 mg/kg, n = 10) or a vehicle at 18 h post-CLP (hypoinflammatory phase) and the SIRT2 modulator enhanced survival (70% AK-7 versus 30% vehicle). AK-7 also enhanced leukocyte adhesion in wild-type mice with diet-induced obesity when administered at 18 h post-CLP (hypoinflammatory stage), confirming the results with ob/ob mice. This experiment suggests that SIRT2 modulators could be beneficial when administered during the hypoinflammatory phase in obese patients with sepsis.

In contrast to the work with SIRT2 modulators, a CLP study with normal, lean mice showed 7-day survival rates for mice with a SIRT2 wild-type (40%), SIRT2 null (10%) versus SIRT2 overexpressing (80%) genotype [168]. Why does this result appear to conflict with the studies using SIRT2 modulators? There are at least three key differences between the experiments. First, as noted earlier, treatment with substrate-selective SIRT2 modulators is not equivalent to the effect of SIRT2 knockout or overexpression. Second, the consequences of SIRT2 modulation in wild-type versus obese mice could be different. There is increased morbidity in human obese patients with sepsis, i.e., prolonged severe disease [169], suggesting that there is an interaction between the two conditions. However, obesity does not appear to cause an increase in sepsis mortality. Third, SIRT2 levels are modulated by dietary obesity [170].

Latent HCMV can reactivate when patients are immunosuppressed. It can also reactivate during times of stress and immune compromise, two features of sepsis. The incidence of HCMV reactivation during sepsis is high, ~30% [171], and, in about half of these patients, the reactivation of additional herpesviruses (EBV, HSV, HHV-6) occurs [172]. Elevated TNF-α during the hyperinflammatory phase, which activates the HCMV major immediate early promoter [173], followed by a hypoinflammatory/immune-suppressed phase, provides a two-pronged mechanism, favoring HCMV reactivation and replication during sepsis. The mortality risk during sepsis is increased ~1.7-fold with HCMV reactivation and reactivation is also associated with a longer requirement for ventilation and prolonged hospitalization [174,175]. Worse outcomes are proportional to the level of circulating viral DNA [174]. Importantly, it is not known whether poor outcomes are a consequence of HCMV activity or simply an indication of the severity of the disease, i.e., severe disease favors HCMV reactivation [176]. An ongoing clinical trial (NCT04706507) designed to test the possible benefits of the direct-acting antiviral, ganciclovir, is currently enrolling patients. If this trial improves outcomes in sepsis patients with reactivated herpesviruses, it will provide a rationale for further investigations with SIRT2-modulating therapeutics, which could potentially block viral replication and spread and also improve outcomes in terms of dysregulated immunity induced by sepsis.

In sum, experiments employing the mouse CLP model raise the possibility that SIRT2 modulators could provide therapeutic benefits in sepsis.

## 7. Some Interesting Questions

### 7.1. Why Does FLS-359 More Potently Inhibit the Production of HCMV Progeny than It Inhibits the Activity of Purified SIRT2?

The FLS-359 IC_50_ for HCMV antiviral activity is 0.47 ± 0.20 µM, whereas the IC_50_ for the inhibition of SIRT2 deacetylation in a biochemical assay is about 3.0 µM [22]. One could suspect that an off-target activity of the drug contributes significantly to the anti-HCMV activity and, of course, an off-target effect remains possible. However, there are many variables that can affect these measurements and resulting interpretations. It is possible that the susceptibility of intracellular SIRT2 to chemical modulators is influenced by phosphorylation and acetylation [122,177,178], or by its numerous associations with other proteins [179]. Alternatively, the inhibition of H3K9 deacetylation (the substrate used in biochemical assays) might be less efficient than the deacetylation of acetylated lysine in other contexts that are highly relevant for antiviral activity. It is also possible that deacetylation activity might not be the key to antiviral activity. A different deacylase activity that is more potently inhibited by the drug might have a greater effect on virus replication. It will be very important to more thoroughly characterize the substrate selectivity across a wide range of lysine modifications targeted by SIRT2 modulators (Table 2), correlating specific sets of enzymatic activities with anti-infective activities.

### 7.2. Which SIRT2-Modulated Processes Must Be Targeted to Generate Anti-Infective Activity?

When nine DNA and RNA viruses were tested, the potency of FLS-359 ranged from 0.3 µM (SARS-CoV-2 in Calu-3 cells) to 6.7 µM (respiratory syncytial virus in MRC-5 fibroblasts) [22]. Part of the reason for these differences is likely trivial, relating to how efficiently the drug enters different cell types, the intracellular half-life once there, and even the fact that the assays were performed in several different laboratories. However, as detailed above, SIRT2 modulators impact numerous cellular processes with the potential to influence viral replication and spread, and it is likely that the perturbation of multiple SIRT2 activities can influence the growth of any one virus and it is also possible that some viruses are more sensitive to the modulation of some SIRT2 targets than others. Further investigation is needed to better understand the extent to which SIRT2-regulated processes influence the growth of a range of different viruses. This understanding could allow SIRT2 modulators to be optimally matched to susceptible pathogens. Indeed, the vision would be a future with a small number of SIRT2 modulators “on the shelf” that could be tested for an optimal match to a new or evolving pathogen.

### 7.3. What Is the Potential for Combination Therapies of SIRT2 Modulators and Direct-Acting Therapeutics?

The therapeutic utility of combining host-targeted SIRT2 modulators with standard-of-care, direct-acting anti-infective drugs holds considerable promise. Combination regimens can potentially enhance potency, reduce the dose of each agent in the cocktail, and block the evolution of drug-resistant variants. Although combinations of other host-targeted compounds with direct-acting agents has been shown to provide a synergistic antiviral effect, for e.g., the mitogen-activated kinase (MEK1/2) inhibitor, ATR-002, with SARS-CoV-2 polymerase or protease inhibitors in cultured cells [180], SIRT2 modulators have not yet been evaluated. This issue is important and deserves exploration.

Although SIRT2 has been most often studied as an anti-infective target among SIRT family members (Table 1), SIRT1 knockdown, knockout, or modulation has also been shown to inhibit the replication of several viruses, including HBV [181,182,183,184,185], HSV-1 [17], HIV [186], and SARS-CoV-2 [187,188]. Further, as noted above, both SIRT1 and SIRT2 have been reported to impact foreign DNA recognition in infected cells. SIRT1 deacetylates IFI16, blocking its cytoplasmic localization [17], whereas SIRT2 deacetylates G3BP1 and blocks its activating interaction with cGAS [77]. These observations raise the possibility that combinations of SIRT1 and SIRT2 modulators might exhibit enhanced antiviral efficacy, since both SIRT1 and SIRT2 inhibit some of the same virus families, and IFI-16 and cGAS cooperate to induce a full innate response to viral DNA [189,190]. This question can be explored by comparing the antiviral properties of combinations of SIRT1 inhibitors, such as EX527 [191], and SIRT2 drugs (Table 2) versus individual drugs. That said, any cross-SIRT isoform binding could result in interference with the expected combined effect. In this case, SIRT1 and 2 dual modulators, such as sirtinol and tenovin-1, which are known to have antiviral activity (Table 2), may be more relevant as a “combination” strategy. Of course, enhanced antiviral activity must be weighed against potentially increased toxicity, if both SIRT1 and SIRT2 are modulated.

## 8. Conclusions

There is clearly a need for a new generation of host-targeted, anti-infective therapeutics. They will bring three principal benefits: broad-spectrum activity, a block to the evolution of resistance, and the potential for rapid deployment in the face of newly emerging pathogens. SIRT2 modulators are highly competitive among host-targeted, broad-spectrum therapeutics, in that they appear to be well-tolerated and likely modulate multiple cell processes that can cooperate to induce an anti-infective environment. They also have the potential advantage that the modification they induce, i.e., hyperacylation, might prove to be relatively long lived, i.e., until eraser activity comes back into balance after the drug is withdrawn. This could reduce the required frequency of dosing. SIRT2 modulators are efficacious in preclinical studies and are poised to provide clinical candidates that can rapidly progress to human safety and efficacy testing.

## Figures and Tables

**Table 1:** Intracellular pathogens reported to be inhibited by SIRT2 modulators.

Infectious Agent	SIRT2 Modulator	Assay (Cell Type)	IC_50_ (µM) ^a^	CC_50_ (µM) ^b^	Reference
**DNA viruses**					
Human cytomegalovirus, strain TB40/E (herpesvirus)	FLS-359	Cell-to-cell spread (MRC-5)	0.5 ± 0.2	>15.8	[22]
Hepatitis B virus genotype D (hepadnavirus)	FLS-359 AGK2	cccDNA establishment (C3A-NTCP) Virus yield (PHH ^c^) Viral RNAs and proteins (Huh7; HepAD38; HepG2-NTCP)	<0.6 4.8 ND ^d^	>10 >10 ND	[34] [22] [35,36]
Epstein–Barr virus, strain Akata (herpesvirus)	FLS-359	EBV gp350 expression (Akata)	3.8	>100	[22]
Herpes simplex 1 (herpesvirus)	AGK2	Virus yield (THP-1 and HeLa)	~5.0	ND	[37]
**RNA viruses**					
SARS-CoV-2 (coronavirus)	FLS-359	Virus yield (Calu-3)	0.3	15.8	[22]
Zika DAK-41525 strain (flavivirus)	FLS-359	Virus yield (HFF ^e^)	0.4	41.6	[22]
Influenza A H1N1 (orthomyxovirus)	FLS-359	Virus yield (dNHBE ^f^)	1.2 ^g^	>100	[22]
OC43 (coronavirus)	FLS-359	CPE reduction (MRC-5)	1.7	>50	[22]
Junin, Candid 1 strain (arenavirus)	FLS-359	Virion antigen reduction (HFF ^e^)	3.2	>25	[22]
Respiratory syncytial virus, long strain (orthopneumovirus)	FLS-359	Virion antigen reduction (MRC-5)	6.7	>12.5	[22]
Hepatitis A virus strain HA11-1299 (picornavirus)	Sirtinol ^h^	Intracellular viral RNA (Huh7)	ND	ND	[38]
Dengue types 1-4 (flavivirus)	Tenovin-1 ^h^	Virus yield (BHK-21)	1.0–3.8	ND	[39]
West Nile virus strain Kunjin (flavivirus); Rift Valley fever virus strain MP12 (bunyavirus)	Tenovin-1 ^h^ Sirtinol ^h^	Intracellular viral RNA (U2OS)	Tenovin-1: 0.4–2.0 Sirtinol: 8.0–40 ^i^	Tenovin-1: >10 Sirtinol: >200	[40]
**Retroviruses**					
HIV NL4-3	AK-1	Reduce viral p24 (T cells; MDMs) Reactivate from latency (J-LAT; primary glial)	ND ^d^	>50	[41]
**Bacteria**					
*Listeria monocytogenes*	AGK2	Reduce bacterial colony forming units per infected cell (Caco2) and in mice	ND ^d^	>20	[18]
*Mycobacterium tuberculosis*	AGK2	Reduce bacterial colony forming units per infected macrophage and in mice	ND ^d^	ND	[42]
*Salmonella typhimurium*	AK-7	Different in vivo versus cultured cell result ^j^	ND	ND	[43]

^a^ Anti-infective 50% inhibitory concentration (IC_50_) in µM; ^b^ 50% cellular cytotoxicity concentration (CC_50_) in µM; ^c^ PHH, primary human hepatocytes; ^d^ anti-infective activity was demonstrated, but IC_50_ was not determined; ^e^ HFF, primary human foreskin fibroblasts; ^f^ dNHBE, differentiated normal human bronchial epithelial cells; ^g^ 1.2 µM = IC_90_; ^h^ SIRT1/2 inhibitor; ^i^ treatment with a combination of EX527 (SIRT1 inhibitor) plus AGK2 (SIRT2 modulator) was not antiviral; ^j^ AK-7 did not reduce intracellular *S. typhimurium* in cultured murine dendritic cells, but reduced bacterial burden in vivo.

**Table 2:** Structures of reported SIRT2 modulators.

Small Molecule	Structure	Chemical Name [Reference] ^a^
**SIRT2 selective**		
FLS-359	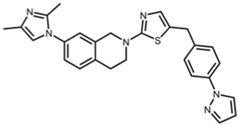	7-(2,4-dimethyl-1H-imidazol-1-yl)-2-(5-{[4-(1H-pyrazol-1-yl)phenyl]methyl}-1,3-thiazol-2-yl)-1,2,3,4-tetrahydroisoquinoline [22]
AGK2	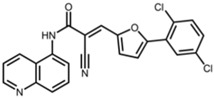	2-cyano-3-[5-(2,5-dichlorophenyl)-2-furanyl]-N-5-quinolinyl-2-propenamide [23]
AK-1	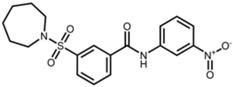	3-(azepan-1-ylsulfonyl)-N-(3-nitrophenyl)benzamide [24]
AK-7	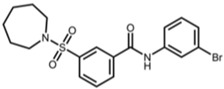	3-(azepan-1-ylsulfonyl)-N-(3-bromophenyl)benzamide [25]
**SIRT1 and SIRT2**		
Sirtinol	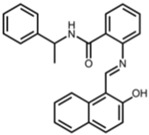	2-[(2-hydroxynaphthalen-1-ylmethylene)amino]-N-(1-phenethyl)benzamide [27]
Tenovin-1	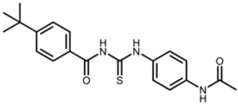	N-[(4-acetamidophenyl)carbamothioyl]-4-tert-butylbenzamide [28]

^a^ First synthesis report of indicated molecule.

**Table 3:** Lysine modifications targeted by SIRT2 in biochemical assays.

Lysine Modification	Peptide ^a^ [Reference]
Acetyl, Propionyl, Butyryl, Hexanoyl, Octanoyl, Decanoyl, Dodecanyl, Myristoyl, Crotonyl	H3K9 [48]
Methacryl	H3K18 [50]
Lipoyl	PDH-E2K259 ^b^ [51]
Benzoyl	H2BK5 [52]
Lactoyl	PKM2K305 ^c^ [53]
4-Oxononanoyl	H3K27 [54]

^a^ Modified peptides are limited to one example and references are limited to one report; ^b^ PDH-E2, pyruvate dehydrogenase E2 component; ^c^ PKM2, pyruvate kinase M2.

## Data Availability

Data is contained within the article.
